# Body mass index and lifetime healthcare utilization

**DOI:** 10.1186/s12913-019-4577-0

**Published:** 2019-10-15

**Authors:** Christina Hansen Edwards, Eline Aas, Jonas Minet Kinge

**Affiliations:** 1 Centre for Fertility and Health, Norwegian Institute of Public Health, Folkehelseinstituttet, Postboks 222 Skøyen, 0213 Oslo, Norway; 2 Department of Health Management and Health Economics, Institute of Health and Society, University of Oslo, Postboks 1089, Blindern, 0317 Oslo, Norway; 3 Centre for Fertility and Health & Centre for Disease Burden, Norwegian Institute of Public Health, Oslo, Norway

**Keywords:** Body mass index, Obesity, Health services, Mortality, Public Policy

## Abstract

**Background:**

Overweight and obesity is a major global public health challenge, and understanding the implications for healthcare systems is essential for policy planning. Past studies have typically found positive associations between obesity and healthcare utilization, but these studies have not taken into consideration that obesity is also associated with early mortality. We examined associations between body mass index (BMI, reported as kg/m^2^) and healthcare utilization with and without taking BMI-specific survival into consideration.

**Methods:**

We used nationally representative data on 33 882 adults collected between 2002 and 2015. We computed BMI- and age-specific primary and secondary care utilization and multiplied the estimated values with gender-, age-, and BMI-specific probabilities of surviving to each age. Then, we summed the average BMI-specific utilization between 18 and 85 years.

**Results:**

During a survival-adjusted lifetime, males with normal weight (BMI: 18.5–24.9) had, on average, 167 primary care, and 77 secondary care contacts. In comparison, males with overweight (BMI: 25.0–29.9), category I obesity (BMI: 30.0–34.9), and category II/III obesity (BMI ≥35.0) had 11%, 41%, and 102% more primary care, and 14%, 29%, and 78% more secondary care contacts, respectively. Females with normal weight had, on average, 210 primary care contacts and 91 secondary care contacts. Females with overweight, category I obesity, and category II/III obesity had 20%, 34%, and 81% more primary care contacts, and 26%, 16%, and 16% more secondary care contacts, respectively.

**Conclusion:**

The positive association between BMI and healthcare utilization was reduced, but not offset, when BMI-specific survival was taken into consideration. Our findings underpin previous research and suggest that interventions to offset the increasing prevalence of overweight, and especially obesity, are warranted.

## Background

Compared with individuals with normal weight, persons with obesity have a greater likelihood of acquiring a range of serious diseases: type-2 diabetes, cardiovascular disease, gallbladder disease, osteoarthritis, hypertension, kidney disease, dyslipidaemia and several cancers [1–3]. Hence, increased healthcare utilization may be needed to manage these conditions. However, in the context of obesity, early mortality may counterbalance increased healthcare utilization due to morbidity. Existing studies that have investigated associations between obesity and healthcare utilization have been done from a non-lifetime perspective, without consideration of early mortality [4]. As a result, it remains unclear whether healthcare utilization over a cumulative lifetime is higher among persons with obesity, compared with persons with normal weight.

Non-lifetime studies have found associations between obesity and physician encounters [5–11], hospitalizations [7, 8, 12], and hospital outpatient visits [6, 7, 12], and specialist visits [9]. A few studies have not found significant associations between obesity and hospitalization [5, 6, 11] or find differing results depending on age [13], or gender [10]. These previous studies vary in terms of methodology, the number of obesity categories assessed, types of healthcare providers included, and the organization of the healthcare system under study. In addition, several of these studies have focused on a specific sub-population (typically females or elderly), rather than a whole population.

The objective of this study was to estimate associations between BMI and utilization of primary and secondary care services from a lifetime cumulative perspective with and without consideration of BMI-specific survival. A few studies have assessed lifetime healthcare costs associated with obesity [14–19] and these studies maintain that obesity is associated with increased healthcare costs, but no studies have assessed lifetime healthcare utilization. The studies evaluating lifetime costs are based on data from the United States, and therefore the findings may not be transferable to settings with a dissimilar healthcare system. Our study was based on data from Norway where the healthcare system is mainly publicly funded, and access to healthcare is universal. As in most countries worldwide, obesity is a major public health challenge in Norway. The proportion of adult obesity has been estimated at 19% among females and 20% among males [20]. In comparison, globally, an estimated 15% of female, and 11% of male adults have obesity [21]. This study contributes to the literature as it is the first to: estimate the effect of obesity on lifetime healthcare utilization, gauge the effect of BMI-specific survival on BMI-specific healthcare utilization, and to use data from a publicly funded healthcare system to assess BMI-specific lifetime healthcare utilization. This study is also the first to estimate BMI-specific healthcare utilization in Norway.

## Methods

To estimate BMI-specific utilization of primary and secondary care services over a lifetime, we computed BMI-, age-, and gender- specific survival probabilities. We then estimated BMI- and gender-specific primary and secondary healthcare utilization from an adult lifetime perspective with and without consideration of survival effects.

### Sample

We used data from the Level of Living Surveys, which are cross-sectional surveys conducted yearly by Statistics Norway. These surveys are intended to provide information about the living conditions of different social groups in Norway. The surveys rotate between three themes: working conditions, living conditions, and health. For this study used the five most recent health-themed studies. These were conducted during the periods 2002-2003, 2005-2006, 2008-2009, 2012-2013 and 2015. The sample populations were selected via stratified random sampling of persons in Norway aged 16 years and above, and are considered to be largely nationally representative. For surveys conducted during the periods 2002-2003, 2005-2006, 2008-2009, and 2012-2013, initial data collection was done via personal face-to-face or telephone interviews. The response rates for these initial interviews were 70%, 70%, 67%, and 58%, respectively. All subjects that had been invited to participate in interviews were later invited to reply to a postal or web-based questionnaire, for which the response rates were 64%, 57%, 50%, and 71%, respectively. In 2015, the survey was conducted via computer-assisted telephone interviews, and no questionnaires were sent. The overall response rate for 2015 was 59%.

The sampling omits individuals in: retirement homes, orphanages, nursing homes, psychiatric institutions, institutions for intellectually disabled, and institutions for drug and alcohol abusers. Detailed information about the sampling procedures and sample representability for each of the study samples have been published in comprehensive reports [22–26]. Data gathered from the surveys had also been linked, using personal ID-numbers, with national registry data on sociodemographic variables such as education and income.

Our dataset was comprised of 33 882 individual-level observations, and included information about healthcare utilization, self-reported height and weight and a range of background variables, including: gender, age, educational level, marital status, smoking status, geographical region of residence, household size, and household income. When reporting weight, pregnant women were asked to provide their pre-pregnancy weight. In terms of healthcare utilization, we used the reported number of primary care contacts, hospital outpatient visits, specialist visits outside of hospital, and hospital inpatient overnight stays; in the past 12 months. For hospital overnight stays, we excluded contacts related to child delivery. In our analyses we combined specialist visits and hospital outpatient and inpatient visits to create a broader outcome category for secondary care. Our data contained information on primary care utilization for all study years and secondary care utilization in 2008-2009, 2012-2013, and 2015.

### BMI computation and classification

We computed the BMI (mass (kg)/ height (m)^2^) of each respondent, and classified each measure according to the World Health Organization’s standard categories: underweight (BMI<18.5), normal weight (BMI ≥18.5 and <25), overweight (BMI ≥25 and <30), category I obesity (BMI ≥30 and <35), and category II/III obesity (a BMI ≥35). To avoid potential bias from response errors we excluded all cases with a BMI<11 and cases with a BMI≥90.

### Calculating BMI-specific survival

BMI-specific survival was estimated by combining gender- and BMI-specific hazard ratios for all-cause mortality, recently estimated by Kjøllesdal and coworkers [27] with national life tables for the Norwegian population in 2015 [28]. To compute these hazard ratios, individuals with BMI measured in early adulthood (18-20 years of age) and in midlife (40-50 years of age) had been followed up until death. If death had not incurred during the follow-up period, individuals were censored at their age at the end of follow-up. The estimated hazard ratios had been adjusted for education and cardiovascular risk factors (smoking, serum total cholesterol, systolic and diastolic blood pressure, current treatment for hypertension, heart rate and height).

**Fig. 1:**
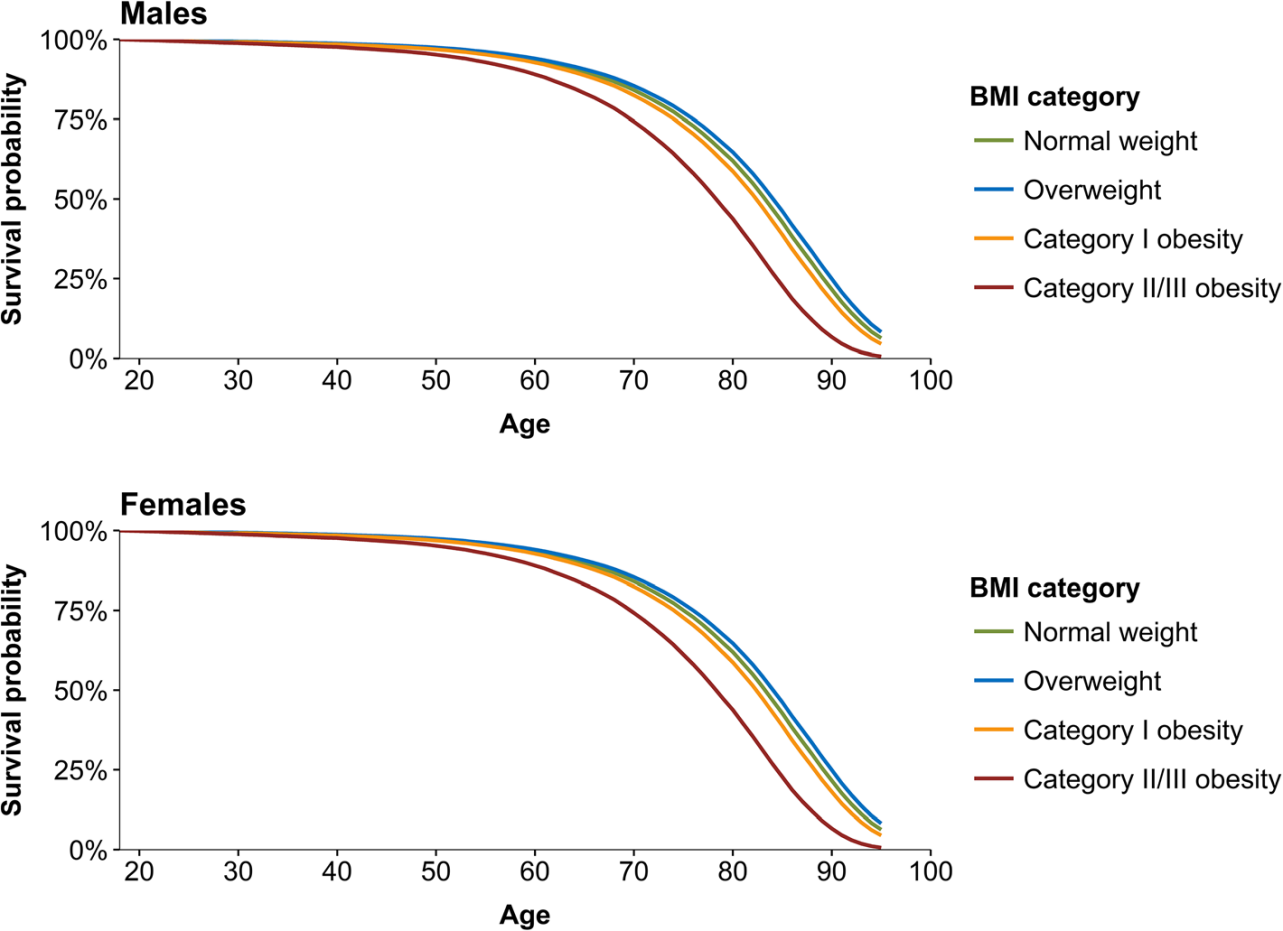
Estimated survival probabilities by age, BMI, and gender

We adapted the hazard ratios to match the proportions of male and female respondents in each BMI-category in our study sample. This was done by multiplying the proportion in each BMI-category in our study sample with the corresponding hazard ratios reported for each BMI-category. For ages 18-29 we applied the hazard ratio estimates based on BMI measured in early adulthood, and for ages 30-85 years we applied the hazard ratios based on BMI measured in midlife. We then computed a mean hazard ratio adjusted for our study sample by summing the calculated products for each BMI-category. Next, we generated BMI specific hazard ratios by dividing the reported BMI-specific hazard ratios by the mean hazard ratio for each BMI-category estimated for our study sample. Finally, we used life tables to generate one-year age-specific survival probabilities (Fig. 1, Additional file 1: Table S1 and Table S2) for males and females in each BMI-category. This was done by multiplying the probability of dying at each age by the BMI-specific adjusted hazard ratios.

### Regression models

#### Dependent and independent variables

BMI-specific healthcare utilization was estimated from two perspectives: a lifetime cumulative perspective, and an annual perspective. In our analyses the dependent variables assessed were: primary care utilization and secondary care utilization, and the independent variables included in the models were: BMI-categories, gender, age (continuous), age^2^, study period (categorical), educational level (categorical), marital status (categorical), geographical region of residence (categorical), and smoking status (categorical). In all our models, we included underweight as a BMI-category, but since the focus of our paper is on overweight and obesity, we do not discuss findings related to underweight. For further information about the variables and categories see Table 1.^1^ We performed our analyses with and without gender stratification, and in the model without stratification, gender was included as a covariate in the model.

**Table 1:** Sociodemographic characteristics of the study sample, by gender and BMI–category, numbers (%)^a^

		Male				Female			
		Normal weight	Overweight	Category I Obesity	Category II/ III Obesity	Normal weight	Overweight	Category I Obesity	Category II/ III Obesity
		(*n*=7 595)	(*n* =7 211)	(*n* =1 516)	(*n* =305)	(*n* =9 810)	(*n* =4 566)	(*n*=1 206)	(*n* =326)
Age	16-24	1 693 (22)	506 (7)	86 (6)	19 (6)	1 671 (17)	325 (7)	68 (6)	17 (5)
	25-44	2 498 (33)	2 453 (34)	551 (36)	125 (41)	3 546 (36)	1 348 (30)	359 (30)	125 (38)
	45-66	2 270 (30)	3 131 (43)	678 (45)	128 (42)	3 237 (33)	1 948 (43)	530 (44)	139 (43)
	67-79	823 (11)	915 (13)	181 (12)	30 (10)	953 (8)	702 (15)	208 (17)	29 (9)
	80+	311 (4)	206 (3)	20 (1)	3 (1)	403 (4)	243 (5)	41 (3)	16 (5)
Marital status	Unmarried	3 600 (47)	2 327 (32)	485 (32)	139 (46)	3 773 (38)	1 191 (26)	334 (28)	94 (29)
	Married	3 278 (43)	4 183 (58)	872 (58)	144 (47)	4 463 (46)	2 472 (54)	604 (50)	169 (52)
	Widow/ widower	198 (3)	167 (2)	38 (3)	6 (2)	658 (7)	439 (10)	145 (12)	26 (8)
	Divorced/ separated	517 (7)	533 (7)	120 (8)	16 (5)	913 (9)	464 (10)	123 (10)	37 (11)
Education	< Upper secondary	1 528 (21)	1 249 (18)	305 (21)	81 (27)	1 806 (19)	978 (22)	291 (25)	81 (25)
	Upper secondary	3 488 (49)	3 815 (54)	842 (57)	151 (51)	4 136 (44)	2 109 (47)	547 (46)	165 (51)
	Higher educ. (short)	1 376 (19)	1 386 (20)	252 (17)	50 (17)	2 778 (30)	1 191 (27)	304 (26)	67 (21)
	Higher educ. (long)	775 (11)	612 (9)	81 (5)	16 (5)	65 (7)	189 (4)	39 (3)	8 (2)
Geographic region	East	3 675 (48)	3 320 (46)	704 (46)	148 (49)	4 939 (50)	2 176 (48)	555 (46)	151 (46)
	West	1 300 (17)	1 241 (17)	256 (17)	37 (12)	1 609 (16)	754 (17)	201 (17)	49 (15)
	South	1 073 (14)	1 075 (15)	210 (14)	46 (15)	1 393 (14)	607 (13)	163 (14)	47 (14)
	Mid & North	1 547 (20)	1 575 (22)	346 (23)	74 (24)	1 869 (19)	1 029 (23)	287 (24)	79 (24)
Smoking status	Daily	1 669 (25)	1 541 (24)	291 (22)	67 (24)	2 006 (23)	945 (23)	251 (23)	62 (21)
	Occasionally	773 (12)	638 (10)	138 (10)	19 (7)	977 (11)	406 (10)	67 (6)	21 (7)
	Non-smoker	4 293 (64)	4 333 (67)	915 (68)	188 (69)	5 731 (66)	2 742 (67)	778 (71)	206 (71)
Study period	Years 2002-2003	1 655 (22)	1 415 (20)	242 (16)	43 (14)	2 061 (21)	878 (19)	219 (18)	45 (14)
	Years 2005-2006	1 571 (21)	1 466 (20)	266 (18)	52 (17)	2 005 (20)	891 (20)	207 (17)	63 (19)
	Years 2008-2009	1 398 (18)	1 372 (19)	297 (20)	53 (17)	1 915 (20)	872 (19)	216 (18)	58 (18)
	Years 2012-2013	1 266 (17)	1 190 (17)	268 (18)	47 (15)	1 626 (17)	805 (18)	193 (16)	54 (17)
	Years 2015-2016	1 705 (22)	1 768 (25)	443 (29)	110 (36)	2 203 (22)	1 120 (25)	371 (31)	106 (33)

^a^Missing data were not included in the calculation of percentages

#### Annual perspective analyses

For each healthcare utilization outcome we had information about whether or not a contact had taken place, and the frequency of contacts. The distributions of our dependent variables were overdispersed and a variety of regression models are available for such situations [29]. For each healthcare utilization outcome, we used Akaike’s information criterion (AIC) to compare: negative binomial models, zero-inflated Poisson models, zero-inflated negative binomial models, logit-Poisson hurdle models, probit-Poisson hurdle models, logit-negative binomial hurdle models, and probit-negative binomial hurdle models. The models with the lowest AIC were selected, and for each outcome the logit-negative binomial hurdle model outperformed the other model variants.

Two-part hurdle models have frequently been used to analyse healthcare utilization [30, 31]. These models regard healthcare utilization as a two-stage process. In our analyses, the first stage of the model, referring to a consumers’ decision to seek healthcare, was modelled as a binary process estimated using a logit model. The second stage is only applied (i.e. the hurdle is only crossed) for those with a positive healthcare utilization. In the second stage, the frequency of contact was modelled using a truncated-at-zero negative binomial model. The advantage of differentiating between the decision to seek healthcare and the frequency of healthcare utilization, is that these processes may be affected by different factors [30]. For instance, a person’s decision to seek healthcare is likely to be influenced by the patients’ subjective need for and ability to seek care. The frequency of consultations, however, might for instance be influenced by the physician and guidelines.

The hurdle regressions were done using the methods described by Deb, Norton, and Manning [32]. Results from the first part of the hurdle model were reported as odds ratios (ORs), and results from the second part of the hurdle model were reported as incidence rate ratios (IRRs). We also report the average expected yearly utilization of primary and secondary care services, for each BMI-category by gender. Regressions were performed in Stata 15 [33] and further analyses were done in R version 3.3.1 [34].

#### Lifetime cumulative perspective analyses

To calculate lifetime utilization with and without survival adjustment, we adapted the method used by Finkelstein and co-workers to estimate lifetime medical costs of obesity [19]. Our lifetime analysis included persons aged 18-85 years. This age restriction was applied for the lifetime analyses because our data did not contain information about individuals in nursing- and retirement homes. To allow for more flexibility in our age-specific estimations, we adapted our hurdle model to include cubic-restricted age-splines with knots at five percentile locations (5%, 27.5%, 50%, 72.5%, and 95%). For each one-year age group we computed BMI-specific average healthcare utilization, while accounting for the age-specific covariate means. This method was applied because some covariate categories would be unrealistic at some ages, and therefore computing age-specific utilization with covariates at the population mean could bias our findings. For instance, in the youngest age groups the probability of being a widow/widower or divorced/separated would be smaller than for an older adult. In the analysis that considered BMI-specific survival, we multiplied the average age- and BMI-specific healthcare utilization with the estimated probabilities of surviving to each age.

Next, we summed the average utilization at each age to obtain a lifetime cumulative utilization estimate. The 95% confidence intervals were calculated using a bootstrap resampling procedure with 500 iterations. The results of the lifetime cumulative perspective analysis can be interpreted as the average expected utilization of primary care services for an 18-year-old, given that he or she remained in the same BMI–category throughout adulthood, and assuming that future healthcare utilization is similar to current utilization.

## Results

In total 676 (2%) of the 33 882 responses in the original dataset were excluded from our analysis. Responses were omitted either because BMI was missing (N=672), or because the calculated BMI was outside our BMI cut-offs (BMI <11 (N=1) and BMI >90 (N=2)). Our final dataset contained information about sociodemographic factors and healthcare utilization (Table 1), for 33 206 respondents. The proportion of male and female respondents was similar, and respondents had an average age of 46.5 years (range: 16–101 years) (Table 1). The average BMI of the respondents was 25.0 kg/m^2^ (range: 11.7–85.7 kg/m^2^), and in total 45% of respondents had overweight or obesity. The proportion of respondents with overweight increased from 43% in 2002 to 49% in 2015, and in the same period the proportion of respondents with obesity increased from 8% to 13 .

In the sample, the mean number of primary care contacts was 2.9 (SD= 4.8) for males, and 3.8 (SD=5.2) for females, and the mean number of secondary care contacts was 1.3 (SD=4.0) for males, and 1.6 (SD=4.5) for females. The crude average number of contacts reported increased with increasing BMI (Table 2).

**Table 2:** Crude mean (SD) number of primary and secondary care contacts per year, by BMI-category and gender

	Normal weight	Overweight	Category I obesity	Category II/III obesity
Mean number of primary care contacts (SD)				
All	3.1 (4.5)	3.4 (4.9)	4.2 (5.8)	6.1 (8.9)
Male	2.6 (4.3)	2.9 (4.8)	3.8 (6.2)	5.5 (6.2)
Female	3.4 (4.6)	4.1 (5.0)	4.7 (5.1)	6.7 (10.7)
Mean number of secondary care contacts (SD)				
All	1.4 (4.0)	1.5 (4.4)	1.7 (4.2)	2.0 (3.9)
Male	1.2 (3.8)	1.6 (3.9)	1.7 (4.8)	1.9 (3.6)
Female	1.5 (4.1)	1.7 (5.1)	1.7 (3.2)	2.1 (4.1)

### Healthcare utilization (annual perspective)

#### Primary care utilization

Compared with persons with normal weight, individuals with overweight and obesity had a significantly (p<0.01) higher probability of primary care contact, and, given that contact had been made, had a significantly (p<0.01) greater number of contacts per year (Additional file 1: Table S3). Increased primary care utilization was seen for males and females with overweight and obesity (Additional file 1: Table S4 and Table S5). Compared with respondents with normal weight, respondents with overweight, category I obesity, and category II/III obesity made, on average, 12%, 34%, and 91% more contacts per year, respectively (Table 3).

**Table 3:** Predicted mean (95% CI) number of primary and secondary care contacts per year, by BMI-category and gender.

	Normal weight	Overweight	Category I obesity	Category II/III obesity
Mean number of primary care contacts (95% CI)				
All	3.3 (3.2; 3.4)	3.7 (3.6; 3.8)	4.5 (4.2; 4.7)	6.3 (5.6; 7.1)
Male	2.9 (2.8; 3.0)	3.2 (3.0; 3.3)	4.0 (3.7; 4.4)	5.9 (5.1; 6.7)
Female	3.7 (3.6; 3.8)	4.4 (4.2; 4.5)	4.8 (4.5; 5.1)	6.8 (5.6; 8.1)
Mean number of secondary care contacts (95% CI)				
All	1.4 (1.2; 1.5)	1.5 (1.4; 1.7)	1.6 (1.4; 1.9)	2.1 (1.7; 2.5)
Male	1.2 (1.1; 1.4)	1.4 (1.2; 1.5)	1.6 (1.3; 1.8)	2.2 (1.6; 2.9)
Female	1.5 (1.3; 1.6)	1.7 (1.5; 1.8)	1.7 (1.4; 1.9)	1.9 (1.5; 2.4)

Estimates were adjusted for age, education marital status, geographic region of residence, smoking status and study period

#### Secondary care utilization

Compared with respondents with normal weight, respondents with overweight and obesity had a significantly (*p*<0.01) higher likelihood of secondary care contact, and respondents with category II/III obesity were more likely to have had a significantly (p<0.01) greater number of contacts (Additional file 1: Table S6). A similar pattern of secondary care utilization was seen for both males and females with overweight and obesity (Additional file 1: Table S7 and S8). The only exception was that females with category II/III obesity were not found to have a higher likelihood of contact, compared with normal weight females. Compared with respondents with normal weight, respondents with overweight, category I obesity, and category II/III obesity made, on average, 13%, 21%, and 51% more contacts per year, respectively (Table 3).

### Lifetime cumulative healthcare utilization

#### Primary care utilization

**Fig. 2:**
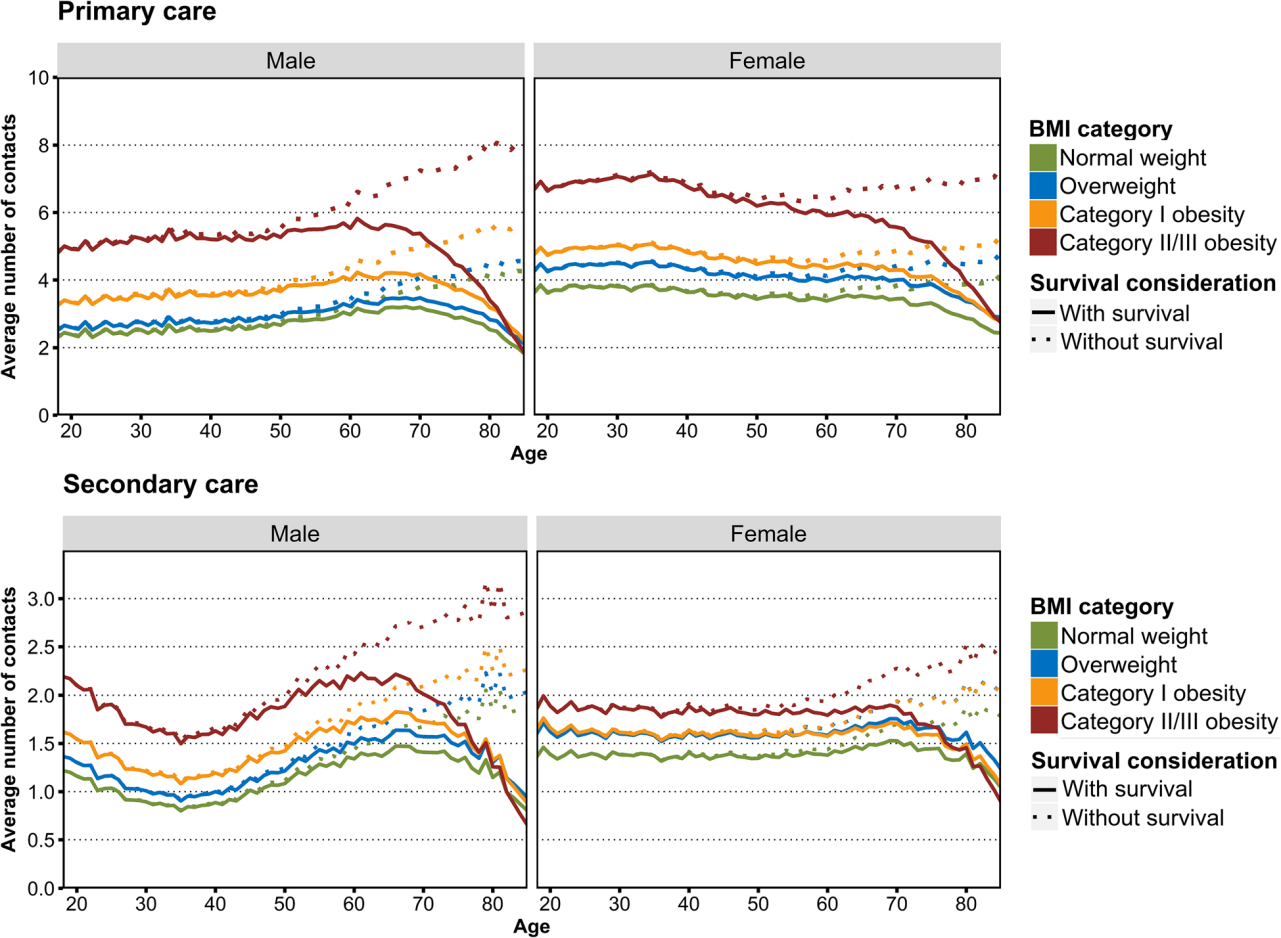
Average age-specific number of primary and secondary care contacts, by BMI and gender estimated from the two-part hurdle model. The analysis was done without consideration of survival (solid lines), and with consideration of BMI-specific survival (dotted lines).

Accounting for survival reduced the mean predicted lifetime cumulative primary care utilization by 12%-17% for males and by 10%-16% for females (Table 4, Fig. 2).

**Table 4:** Predicted mean (95% CI) number of primary care contacts over a lifetime, by BMI–category and gender

Gender	BMI–category	Mean (95% CI) without survival	Mean (95% CI) with survival	Mean (%) reduction due to survival
Male	Normal weight	193 (185; 201)	167 (160; 173)	26 (13%)
	Overweight	211 (202; 221)	185 (178; 193)	26 (12%)
	Category I obesity	272 (249; 296)	235 (216; 254)	37 (14%)
	Category II/III obesity	406 (348; 464)	338 (289; 387)	68 (17%)
Female	Normal weight	236 (229; 244)	210 (204; 216)	26 (11%)
	Overweight	281 (270; 293)	253 (244; 264)	28 (10%)
	Category I obesity	317 (295; 338)	282 (264; 301)	35 (11%)
	Category II/III obesity	450 (364; 535)	380 (310; 450)	70 (16%)

Estimates were adjusted for age, education marital status, geographic region of residence, smoking status and study period. The lifetime utilization was estimates based on participants between 18 and 85 years of age

After considering survival, males with overweight, category I obesity, and category II/III obesity made contact with a primary care provider on average 11%, 41%, and 102% more often than males with normal weight, respectively (Table 4). Females with overweight, category I obesity, and category II/III obesity made contact with a primary care provider, on average, 20%, 34%, and 81% more often compared with females with normal weight, respectively (Table 4). The confidence intervals suggest that both before and after taking survival into consideration, males and females with overweight and obesity had a significantly higher primary care utilization, compared with their normal weight counterparts.

#### Secondary care utilization

Accounting for survival reduced the mean predicted lifetime cumulative secondary care utilization by 13%-17% for males and 11%-15% for females (Table 5, Fig. 2).

**Table 5:** Predicted mean (with 95% confidence intervals) number of secondary care contacts over a lifetime, by BMI–category and gender

Gender	BMI–category	Mean (95% CI) without survival	Mean (95% CI) with survival	Mean (%) reduction due to survival
Male	Normal weight	92 (82; 103)	77 (69; 86)	15 (16%)
	Overweight	101 (91; 112)	88 (79; 98)	13 (13%)
	Category I obesity	115 (94; 136)	99 (81; 117)	16 (14%)
	Category II/III obesity	166 (112; 219)	137 (94; 180)	29 (17%)
Female	Normal weight	103 (94; 113)	91 (84; 98)	12 (12%)
	Overweight	119 (105; 133)	106 (94; 117)	13 (11%)
	Category I obesity	119 (101; 136)	106 (91; 121)	13 (11%)
	Category II/III obesity	136 (101; 171)	115 (87; 144)	21 (15%)

Estimates were adjusted for age, education marital status, geographic region of residence, smoking status and study period. The lifetime utilization was estimates based on participants between 18 and 85 years of age

After accounting for survival, males with overweight, category I obesity, and category II/III obesity made, on average, 14%, 29%, and 78% (Table 5) more contacts than normal weight males, respectively. Females with overweight, category I obesity, and category II/III obesity made, on average, 16%, 16%, and 26% (Table 5) more contacts than normal weight females. After taking survival into consideration there was overlap between the confidence intervals of nearly all the BMI-categories within each gender strata. The only exception was that the confidence intervals of males with category II/III obesity and normal weight did not overlap.

## Discussion

The study has quantified the BMI-specific consequences of overlooking survival-adjustment when estimating the association between BMI and healthcare utilization. After consideration of survival, we found a positive association between overweight and obesity and primary care utilization in both males and females; secondary care utilization was only found to be significantly higher for males with category II/III obesity, compared with normal weight males. Nevertheless, there was a tendency for secondary care utilization to increase with BMI. Our estimates suggest that during a lifetime, an 18-year-old male with a BMI≥35, can be expected to make 171 (102%) more primary care, and 60 (78%) more secondary care contacts, compared with a normal weight male. An 18-year old female with a BMI≥35 can be expected to make 170 (81%) more primary care, and 24 (26%) more secondary care contacts, compared with a normal weight female.

Studies that have assessed associations between excess BMI and healthcare utilization without a lifetime perspective have typically found positive associations between obesity and primary care utilization [5–11]. These findings correspond well with the results from our annual perspective analyses. For both males and females we found positive associations between overweight and obesity and the likelihood of primary care utilization. Most previous studies [6–9, 12] have found positive associations between obesity and secondary care utilization, but a few do not find such an association [5, 11]. Our results for secondary care utilization are varied, and differ between males and females.

The present study was the first to compute healthcare utilization for different BMI-categories over a lifetime and was the first to consider the effect of BMI-specific survival on healthcare utilization. Our study also had some further advantages: I) We estimated healthcare utilization associated with obesity in a public healthcare setting II) we used a nationally representative sample of male and female adults; III) we examined differences in primary and secondary care utilization between respondents with normal weight and three different categories related to excess BMI (overweight, and category I and II/III obesity). Moreover, the survey response rate was reasonably high, with yearly response rates varying from 50–71%.

This study had some limitations. First, although sampling for this study was undertaken strategically to select a sample that can be considered representative of the population, any questionnaire-based study may be subject to selection bias. For instance, a potential problem was that persons in nursing homes and persons above 85 years of age were not included in the sample. This could be problematic as these groups of individuals may have different healthcare seeking behaviour compared with the rest of the population. Also, since obesity is associated with a shorter life expectancy, we may have captured a smaller proportion of lifetime costs for persons with normal weight, than for persons with obesity. Second, respondents reported healthcare utilization during the past year, and responses may have been subject to recall bias. Third, with the exception of the data that was registry-based (such as data on income and education), data were self-reported and may have been subject to response bias. In particular, self-reported height and weight measures tend to result in an underestimation of true BMI; the extent of bias differs between sexes, and increases with age [35, 36]. Errors in self-reported healthcare utilization data have been shown to increase with the number of healthcare encounters experienced [37]. Also, using self-reported data on healthcare utilization is likely to lead to an exclusion of end-of-life utilization. Fourth, BMI is known to be an imperfect measure of adiposity. Ideally, hip and waist measurements or other body composition measurements should supplement BMI measures, but such data were not available. Fifth, although hurdle models have been shown to be superior in settings in which general practitioners act as gatekeepers to specialist care [38], hurdle models have been criticized for not allowing distinction between high and low frequency users of healthcare [39]. Lastly, our lifetime utilization estimates do not take into account that an 18-year-old with a particular BMI may change BMI–category over a lifetime. However, a review by Simmonds et al. found that 80% of persons with adolescent obesity will have obesity in adulthood [40].

Population BMI is increasing, and although some studies have found indications that BMI has been increasing at a slower rate [41], predictions suggest that the proportion of the population with obesity is likely to continue to rise [42]. If the effects of increased BMI on healthcare utilization described in this study are in fact causal, increasing youth obesity is likely to result in increased healthcare costs in the future. In the short term, efficient planning to tackle increased demands for healthcare is warranted to reduce future economic and organizational burdens on the healthcare system. At the same time, it is essential to identify and implement effective interventions to prevent overweight, and especially obesity. In the long-run, as more data become available and new methods develop, it will be important to differentiate between obesity-related and obesity-caused drivers of healthcare utilization and costs, and to implement appropriate prevention strategies accordingly.

Our associations cannot be interpreted as causal, because our results are likely biased due to omitted variables (such as lifestyle factors [43], genetic factors [44], and time lived with overweight or obesity [45]), measurement error in covariates, and reverse causality. Despite these issues, it seems plausible that there is a positive causal association between obesity and healthcare utilization, however the amount and type of healthcare utilization owing specifically to obesity is difficult to ascertain. To reduce bias, a randomized study would be needed, but randomization in this context would be problematic. Future studies may want to explore the application of genetic information in instrumental variable analyses [46] as a way of reducing bias. Moreover, since treatment for a particular disease may depend on BMI, future studies should explore disease-specific healthcare utilization by BMI. Finally, this study suggests that healthcare utilization increases with BMI, and the implications of this increased demand on patients and providers should be investigated.

## Conclusion

The effect of excess BMI on healthcare utilization was reduced, but not completely offset, when BMI-specific survival was taken into consideration. Future studies should attempt to account for BMI-specific survival when assessing the health and/or economic consequences of obesity. As the BMI of populations worldwide continues to rise, it is becoming increasingly important to understand the consequences. In this study, both overweight and obesity, but especially obesity, was associated with increased utilization of healthcare. As a result, healthcare payers and providers are likely to benefit from the implementation of interventions to combat obesity.

## Supplementary information

**Additional file 1.** Body mass index and lifetime healthcare utilization. Additional information. This file contains the estimated BMI-and one-year age-specific survival probabilities for males (**Table S1.**) and females (**Table S2.**) As well as results from the hurdle regressions estimating the likelihood and frequency of primary care (**Tables S3** – **S5.**) and secondary care (**Tables S6** – **S8.**) contacts, for both males and females, and stratified by gender.

## Data Availability

The data that support the findings of this study are available from the Norwegian Centre for Research Data (NSD), but restrictions apply to the availability of these data, which were used under license for the current study, and so are not publicly available.

## Abbreviations

AIC: Akaike’s information criterion
BMI: Body mass index
IRR: Incidence rate ratios
OR: Odds ratio

## Footnotes

1 We explored the inclusion of sample weights (based on gender, 5-year age-groups, four education levels, and seven geographical regions), but this did not make a significant difference to the results. As a result we left the weights out to ease the interpretation of our findings.
