# Supplementary material for: Body mass index and lifetime healthcare utilization
Source: BMC Health Serv Res. 2019 Oct 15;19:696. doi: 10.1186/s12913-019-4577-0 (PMC6794833; doi:10.1186/s12913-019-4577-0)
Supplement: Supplementary file 1 — Additional file 1. Body mass index and lifetime healthcare utilization. Additional information. This file contains the estimated BMI-and one-year age-specific survival probabilities for males (Table S1.) and females (Table S2.) As well as results from the hurdle regressions estimating the likelihood and frequency of primary care (Tables S3 – S5.) and secondary care (Tables S6 – S8.) contacts, for both males and females, and stratified by gender. [file 12913_2019_4577_MOESM1_ESM.docx]

**Body mass index and lifetime healthcare utilization**

Christina Hansen Edwards^1*^, Eline Aas^2^, Jonas Minet Kinge^1,2^

^1^Norwegian Institute of Public Health, Oslo, Norway.

^2^Department of Health Management and Health Economics, Institute of Health and Society, University of Oslo, Oslo, Norway.

Correspondence to: christina.hansen.edwards@fhi.no

# Additional information

**Overview of tables**

[**Table S1: Estimated BMI- and age-specific survival probabilities for males.** 3](#_Toc20397176)

[**Table S2: Estimated BMI- and age-specific survival probabilities for females.** 5](#_Toc20397177)

[**Table S3: Results of hurdle regressions on primary care consultations; adjusted for gender, age, education, marital status, geographical region, smoking status, and study period.** 7](#_Toc20397178)

[**Table S4: Results of hurdle regressions on primary care consultations among males; adjusted for, age, education, marital status, geographical region, smoking status, and study period.** 8](#_Toc20397179)

[**Table S5: Results of hurdle regressions on primary care consultations among females; adjusted for, age, education, marital status, geographical region, smoking status, and study period.** 9](#_Toc20397180)

[**Table S6: Results of hurdle regressions on secondary care consultations; adjusted for gender, age, education, marital status, geographical region, smoking status, and study period.** 10](#_Toc20397181)

[**Table S7: Results of hurdle regressions on secondary care consultations among males; adjusted for age, education, marital status, geographical region, smoking status, and study period.** 11](#_Toc20397182)

[**Table S8: Results of hurdle regressions on secondary care consultations among females; adjusted for age, education, marital status, geographical region, smoking status, and study period.** 12](#_Toc20397183)

**Table S1: Estimated BMI- and age-specific survival probabilities for males.**

|  | Estimated survival probabilities by BMI category | | | | |
| --- | --- | --- | --- | --- | --- |
| Age | Underweight | Normal | Overweight | Obese I | Obese II and III |
| 18 | 1.000000000 | 1.000000000 | 1.000000000 | 1.000000000 | 1.000000000 |
| 19 | 0.999566370 | 0.999605791 | 0.999523007 | 0.999294365 | 0.999183987 |
| 20 | 0.999081770 | 0.999165228 | 0.998989970 | 0.998506004 | 0.998272407 |
| 21 | 0.998731280 | 0.998846574 | 0.998604466 | 0.997935989 | 0.997613382 |
| 22 | 0.998194112 | 0.998358183 | 0.998013656 | 0.997062567 | 0.996603661 |
| 23 | 0.997614505 | 0.997831181 | 0.997376204 | 0.996120458 | 0.995514685 |
| 24 | 0.997151275 | 0.997409971 | 0.996866773 | 0.995367785 | 0.994644804 |
| 25 | 0.996592687 | 0.996902033 | 0.996252502 | 0.994460436 | 0.993596287 |
| 26 | 0.995963933 | 0.996330260 | 0.995561108 | 0.993439470 | 0.992416642 |
| 27 | 0.995486640 | 0.995896199 | 0.995036299 | 0.992664754 | 0.991521664 |
| 28 | 0.994873405 | 0.995338483 | 0.994362045 | 0.991669682 | 0.990372263 |
| 29 | 0.994230923 | 0.994754135 | 0.993655678 | 0.990627555 | 0.989168699 |
| 30 | 0.982992013 | 0.993137409 | 0.993753274 | 0.992385159 | 0.988291143 |
| 31 | 0.981617543 | 0.992579716 | 0.993245458 | 0.991766589 | 0.987342142 |
| 32 | 0.980043656 | 0.991940574 | 0.992663449 | 0.991057722 | 0.986254976 |
| 33 | 0.978214892 | 0.991197215 | 0.991986499 | 0.990233328 | 0.984991118 |
| 34 | 0.976098365 | 0.990335923 | 0.991202100 | 0.989278224 | 0.983527531 |
| 35 | 0.974389275 | 0.989639530 | 0.990567828 | 0.988506053 | 0.982344885 |
| 36 | 0.972556439 | 0.988891931 | 0.989886874 | 0.987677169 | 0.981075914 |
| 37 | 0.970848685 | 0.988194566 | 0.989251634 | 0.986904045 | 0.979892845 |
| 38 | 0.969185213 | 0.987514569 | 0.988632175 | 0.986150234 | 0.978739819 |
| 39 | 0.967245801 | 0.986720958 | 0.987909172 | 0.985270543 | 0.977394803 |
| 40 | 0.965828024 | 0.986140106 | 0.987379959 | 0.984626744 | 0.976410933 |
| 41 | 0.962689812 | 0.984853274 | 0.986207470 | 0.983200553 | 0.974232160 |
| 42 | 0.959959120 | 0.983731362 | 0.985185127 | 0.981957321 | 0.972334381 |
| 43 | 0.957413861 | 0.982683857 | 0.984230488 | 0.980796686 | 0.970563899 |
| 44 | 0.954540076 | 0.981499264 | 0.983150812 | 0.979484314 | 0.968563229 |
| 45 | 0.951290507 | 0.980157358 | 0.981927623 | 0.977997856 | 0.966298813 |
| 46 | 0.948009171 | 0.978799563 | 0.980689798 | 0.976494024 | 0.964009813 |
| 47 | 0.944879059 | 0.977501662 | 0.979506427 | 0.975056747 | 0.961823937 |
| 48 | 0.940601184 | 0.975724324 | 0.977885732 | 0.973088836 | 0.958833434 |
| 49 | 0.936410906 | 0.973978642 | 0.976293643 | 0.971156364 | 0.955899994 |
| 50 | 0.930908878 | 0.971680343 | 0.974197220 | 0.968612644 | 0.952042852 |
| 51 | 0.925651073 | 0.969476294 | 0.972186339 | 0.966173873 | 0.948350096 |
| 52 | 0.919656576 | 0.966954885 | 0.969885444 | 0.963384643 | 0.944132443 |
| 53 | 0.913351243 | 0.964292387 | 0.967455228 | 0.960440183 | 0.939687031 |
| 54 | 0.904610343 | 0.960586197 | 0.964071532 | 0.956342746 | 0.933511158 |
| 55 | 0.896745054 | 0.957231988 | 0.961008127 | 0.952636021 | 0.927937127 |
| 56 | 0.887761569 | 0.953380809 | 0.957489729 | 0.948381737 | 0.921553152 |
| 57 | 0.878175253 | 0.949246314 | 0.953711124 | 0.943816512 | 0.914719190 |
| 58 | 0.868130119 | 0.944885635 | 0.949724241 | 0.939003845 | 0.907533655 |
| 59 | 0.857064197 | 0.940048562 | 0.945299964 | 0.933668116 | 0.899589234 |
| 60 | 0.844040539 | 0.934311750 | 0.940050302 | 0.927343476 | 0.890201502 |
| 61 | 0.830636305 | 0.928352781 | 0.934594334 | 0.920778352 | 0.880492742 |
| 62 | 0.814621341 | 0.921164439 | 0.928008963 | 0.912864394 | 0.868834379 |
| 63 | 0.798711387 | 0.913939220 | 0.921385160 | 0.904916664 | 0.857181133 |
| 64 | 0.782293042 | 0.906394251 | 0.914463301 | 0.896624427 | 0.845080478 |
| 65 | 0.763464869 | 0.897633194 | 0.906419765 | 0.887004475 | 0.831112500 |
| 66 | 0.744778573 | 0.888809840 | 0.898311917 | 0.877326520 | 0.817142682 |
| 67 | 0.723678834 | 0.878697319 | 0.889011143 | 0.866246646 | 0.801244606 |
| 68 | 0.701629160 | 0.867945146 | 0.879111818 | 0.854480846 | 0.784479042 |
| 69 | 0.674995043 | 0.854713204 | 0.866915835 | 0.840021234 | 0.764028313 |
| 70 | 0.647413430 | 0.840687000 | 0.853969762 | 0.824719770 | 0.742588288 |
| 71 | 0.618460116 | 0.825587883 | 0.840012471 | 0.808278074 | 0.719781641 |
| 72 | 0.587744804 | 0.809121159 | 0.824765941 | 0.790383240 | 0.695232248 |
| 73 | 0.556455565 | 0.791822182 | 0.808719491 | 0.771626060 | 0.669814746 |
| 74 | 0.523811649 | 0.773166975 | 0.791380984 | 0.751446938 | 0.642829696 |
| 75 | 0.486718956 | 0.751178902 | 0.770900468 | 0.727725819 | 0.611568476 |
| 76 | 0.450890580 | 0.728971749 | 0.750161402 | 0.703845492 | 0.580651949 |
| 77 | 0.413260457 | 0.704538803 | 0.727281127 | 0.677659716 | 0.547372423 |
| 78 | 0.373270491 | 0.677158847 | 0.701561092 | 0.648427450 | 0.510997100 |
| 79 | 0.333591054 | 0.648249848 | 0.674305892 | 0.617699975 | 0.473692959 |
| 80 | 0.296650187 | 0.619420437 | 0.647016636 | 0.587207414 | 0.437669420 |
| 81 | 0.255622936 | 0.585016038 | 0.614313810 | 0.551004548 | 0.396100306 |
| 82 | 0.217297252 | 0.549790429 | 0.580653166 | 0.514177330 | 0.355316098 |
| 83 | 0.178782356 | 0.510654799 | 0.543040567 | 0.473550676 | 0.312066095 |
| 84 | 0.142204845 | 0.468696530 | 0.502437036 | 0.430361037 | 0.268219806 |
| 85 | 0.110928220 | 0.427296765 | 0.462051191 | 0.388165932 | 0.227706928 |
| 86 | 0.081470384 | 0.381725712 | 0.417208574 | 0.342214415 | 0.186179808 |
| 87 | 0.059421616 | 0.340236362 | 0.375943766 | 0.300928065 | 0.151576796 |
| 88 | 0.041333765 | 0.298643013 | 0.334121511 | 0.260093415 | 0.119890462 |
| 89 | 0.026634713 | 0.255991211 | 0.290697408 | 0.218861134 | 0.090610819 |
| 90 | 0.016237528 | 0.215858967 | 0.249225794 | 0.180775603 | 0.066319866 |
| 91 | 0.008842395 | 0.176377180 | 0.207743660 | 0.144073622 | 0.045577110 |
| 92 | 0.004475908 | 0.141398344 | 0.170252220 | 0.112358207 | 0.030120799 |
| 93 | 0.002060037 | 0.110747844 | 0.136668606 | 0.085323540 | 0.018955873 |
| 94 | 0.000799034 | 0.083522274 | 0.106094635 | 0.062040829 | 0.010987281 |
| 95 | 0.000304154 | 0.062747444 | 0.082080332 | 0.044911682 | 0.006314004 |

**Table S2: Estimated BMI- and age-specific survival probabilities for females.**

|  | Estimated survival probabilities by BMI category | | | | |
| --- | --- | --- | --- | --- | --- |
| Age | Underweight | Normal | Overweight | Obese I | Obese II and III |
| 18 | 1.000000000 | 1.000000000 | 1.000000000 | 1.000000000 | 1.000000000 |
| 19 | 0.999766992 | 0.999773779 | 0.999751157 | 0.999624473 | 0.999504576 |
| 20 | 0.999592979 | 0.999604833 | 0.999565320 | 0.999344064 | 0.999134683 |
| 21 | 0.999447993 | 0.999464068 | 0.999410485 | 0.999110456 | 0.998826553 |
| 22 | 0.999303963 | 0.999324231 | 0.999256673 | 0.998878409 | 0.998520506 |
| 23 | 0.999076728 | 0.999103610 | 0.999014006 | 0.998512341 | 0.998037735 |
| 24 | 0.998856090 | 0.998889392 | 0.998778387 | 0.998156950 | 0.997569098 |
| 25 | 0.998615871 | 0.998656162 | 0.998521863 | 0.997770072 | 0.997058999 |
| 26 | 0.998380382 | 0.998427523 | 0.998270394 | 0.997390868 | 0.996559081 |
| 27 | 0.998066471 | 0.998122741 | 0.997935186 | 0.996885455 | 0.995892857 |
| 28 | 0.997618168 | 0.997687471 | 0.997456479 | 0.996163802 | 0.994941744 |
| 29 | 0.997403453 | 0.997478995 | 0.997227208 | 0.995818260 | 0.994486438 |
| 30 | 0.995230343 | 0.997035045 | 0.997212717 | 0.996472606 | 0.994609739 |
| 31 | 0.994776168 | 0.996752437 | 0.996947018 | 0.996136493 | 0.994096643 |
| 32 | 0.994272620 | 0.996439053 | 0.996652380 | 0.995763796 | 0.993527804 |
| 33 | 0.993721320 | 0.996095885 | 0.996329732 | 0.995355703 | 0.992905062 |
| 34 | 0.993263190 | 0.995810652 | 0.996061551 | 0.995016528 | 0.992387602 |
| 35 | 0.992893452 | 0.995580412 | 0.995845071 | 0.994742761 | 0.991970005 |
| 36 | 0.992291884 | 0.995205756 | 0.995492801 | 0.994297296 | 0.991290605 |
| 37 | 0.991499038 | 0.994711859 | 0.995028403 | 0.993710095 | 0.990395248 |
| 38 | 0.990788672 | 0.994269208 | 0.994612179 | 0.993183870 | 0.989593120 |
| 39 | 0.990080358 | 0.993827716 | 0.994197033 | 0.992659068 | 0.988793383 |
| 40 | 0.989253811 | 0.993312390 | 0.993712447 | 0.992046551 | 0.987860240 |
| 41 | 0.988193756 | 0.992651269 | 0.993090743 | 0.991260820 | 0.986663605 |
| 42 | 0.987156385 | 0.992004033 | 0.992482072 | 0.990491688 | 0.985492740 |
| 43 | 0.986475268 | 0.991578901 | 0.992082255 | 0.989986552 | 0.984724079 |
| 44 | 0.985602564 | 0.991034045 | 0.991569830 | 0.989339215 | 0.983739296 |
| 45 | 0.984144228 | 0.990123254 | 0.990713224 | 0.988257227 | 0.982093859 |
| 46 | 0.982694181 | 0.989217132 | 0.989860962 | 0.987180974 | 0.980458090 |
| 47 | 0.980470276 | 0.987826656 | 0.988553064 | 0.985529714 | 0.977949832 |
| 48 | 0.979048549 | 0.986936970 | 0.987716144 | 0.984473449 | 0.976346793 |
| 49 | 0.977557213 | 0.986003212 | 0.986837718 | 0.983365050 | 0.974665588 |
| 50 | 0.975314483 | 0.984598176 | 0.985515866 | 0.981697531 | 0.972137827 |
| 51 | 0.972870304 | 0.983065602 | 0.984073904 | 0.979879141 | 0.969383841 |
| 52 | 0.970015554 | 0.981273884 | 0.982387962 | 0.977753907 | 0.966168298 |
| 53 | 0.967583144 | 0.979745535 | 0.980949683 | 0.975941696 | 0.963429523 |
| 54 | 0.964442504 | 0.977770307 | 0.979090686 | 0.973600300 | 0.959894474 |
| 55 | 0.960610561 | 0.975357327 | 0.976819422 | 0.970741101 | 0.955583140 |
| 56 | 0.955869214 | 0.972367182 | 0.974004472 | 0.967199669 | 0.950251407 |
| 57 | 0.950853512 | 0.969198069 | 0.971020491 | 0.963448467 | 0.944614807 |
| 58 | 0.946235853 | 0.966274624 | 0.968267285 | 0.959990205 | 0.939429092 |
| 59 | 0.941099742 | 0.963016935 | 0.965198742 | 0.956138767 | 0.933664820 |
| 60 | 0.935718875 | 0.959596946 | 0.961976669 | 0.952098049 | 0.927630156 |
| 61 | 0.929459359 | 0.955609835 | 0.958219490 | 0.947390464 | 0.920615349 |
| 62 | 0.922072018 | 0.950892329 | 0.953772924 | 0.941824917 | 0.912343902 |
| 63 | 0.914173243 | 0.945832913 | 0.949002666 | 0.935861624 | 0.903509056 |
| 64 | 0.906786369 | 0.941085897 | 0.944525517 | 0.930272228 | 0.895256087 |
| 65 | 0.898340616 | 0.935641663 | 0.939389233 | 0.923868033 | 0.885830115 |
| 66 | 0.889896572 | 0.930179141 | 0.934233896 | 0.917449430 | 0.876417605 |
| 67 | 0.880093202 | 0.923814461 | 0.928225017 | 0.909979113 | 0.865503391 |
| 68 | 0.869535622 | 0.916931195 | 0.921723856 | 0.901910698 | 0.853766583 |
| 69 | 0.857172083 | 0.908833409 | 0.914072151 | 0.892432189 | 0.840043867 |
| 70 | 0.843127270 | 0.899584163 | 0.905327743 | 0.881624216 | 0.824484375 |
| 71 | 0.828284360 | 0.889747634 | 0.896022371 | 0.870152443 | 0.808076442 |
| 72 | 0.810636268 | 0.877972679 | 0.884875856 | 0.856448842 | 0.788613152 |
| 73 | 0.793491721 | 0.866439339 | 0.873949274 | 0.843060632 | 0.769758891 |
| 74 | 0.775562880 | 0.854279660 | 0.862420105 | 0.828981052 | 0.750097691 |
| 75 | 0.755275348 | 0.840399758 | 0.849248671 | 0.812953105 | 0.727916975 |
| 76 | 0.732495280 | 0.824655951 | 0.834293666 | 0.794829847 | 0.703098388 |
| 77 | 0.708300802 | 0.807737588 | 0.818204544 | 0.775425157 | 0.676845744 |
| 78 | 0.680765204 | 0.788233699 | 0.799633315 | 0.753144000 | 0.647100879 |
| 79 | 0.651902725 | 0.767476661 | 0.779839517 | 0.729542729 | 0.616087165 |
| 80 | 0.616977793 | 0.741938356 | 0.755446811 | 0.700654256 | 0.578775860 |
| 81 | 0.581581123 | 0.715499977 | 0.730142253 | 0.670943225 | 0.541239791 |
| 82 | 0.543024842 | 0.686037545 | 0.701880813 | 0.638066262 | 0.500677725 |
| 83 | 0.502511649 | 0.654246941 | 0.671307526 | 0.602880768 | 0.458451664 |
| 84 | 0.460762500 | 0.620485784 | 0.638744483 | 0.565859269 | 0.415394982 |
| 85 | 0.419853896 | 0.586268661 | 0.605633911 | 0.528725669 | 0.373703792 |
| 86 | 0.375150023 | 0.547496700 | 0.567984419 | 0.487115599 | 0.328723738 |
| 87 | 0.327798795 | 0.504574507 | 0.526127749 | 0.441671302 | 0.281820497 |
| 88 | 0.282052870 | 0.460837961 | 0.483259254 | 0.396113231 | 0.237361143 |
| 89 | 0.237137363 | 0.415256509 | 0.438328062 | 0.349489587 | 0.194632321 |
| 90 | 0.192429263 | 0.366629584 | 0.390079154 | 0.300788174 | 0.153151561 |
| 91 | 0.150691748 | 0.317237529 | 0.340681060 | 0.252567052 | 0.115600483 |
| 92 | 0.113978075 | 0.269231360 | 0.292220511 | 0.207085409 | 0.083762648 |
| 93 | 0.082655997 | 0.223276757 | 0.245334647 | 0.165022449 | 0.057741578 |
| 94 | 0.057043840 | 0.180304376 | 0.200950013 | 0.127227315 | 0.037515767 |
| 95 | 0.038628511 | 0.144150811 | 0.163074305 | 0.096869382 | 0.023824927 |

**Table S3: Results of hurdle regressions on primary care consultations; adjusted for gender, age, education, marital status, geographical region, smoking status, and study period.**

| Variables | Logit  OR (SE) | GLM  IRR (SE) |
| --- | --- | --- |
| Underweight | 0.91 (0.12) | 1.16 (0.13) |
| Overweight | 1.23 (0.05)*** | 1.14 (0.03)*** |
| Category I obesity | 1.67 (0.12)*** | 1.42 (0.06)*** |
| Category II/III obesity | 2.41 (0.41)*** | 2.20 (0.17)*** |
| Female | 1.67 (0.06)*** | 1.31 (0.03)*** |
| Age | 0.98 (0.01)*** | 1.00 (0.00) |
| Age^2 | 1.00 (0.00)*** | 1.00 (0.00)* |
| Study period 2005-2006 | 0.78 (0.03)*** | 1.08 (0.04)* |
| Study period 2008-2009 | 1.69 (0.08)*** | 1.04 (0.04) |
| Study period 2012-2013 | 1.64 (0.12)*** | 0.97 (0.05) |
| Study period 2015-2016^1^ | 95.94 (22.26)*** | 1.05 (0.04) |
| Higher education. long | 0.77 (0.06)*** | 0.72 (0.04)*** |
| Higher education. short | 0.94 (0.05) | 0.75 (0.03)*** |
| Upper secondary education | 0.99 (0.05) | 0.84 (0.03)*** |
| Divorced /separated | 1.06 (0.07) | 1.31 (0.06)*** |
| Unmarried | 0.94 (0.05) | 1.09 (0.04)*** |
| Widow/widower | 1.01 (0.10) | 1.00 (0.05) |
| Mid-North Norway | 0.94 (0.04) | 0.89 (0.03)*** |
| Southern Norway | 0.88 (0.04)** | 0.99 (0.04) |
| Western Norway | 0.90 (0.04)** | 0.96 (0.03) |
| Smoking daily | 1.17 (0.05)*** | 1.25 (0.04)*** |
| Smoking occasionally | 1.07 (0.06) | 0.96 (0.04) |
| Constant | 2.21 (0.34)*** | 1.84 (0.21)*** |
| Number of observations | 26,419 | 21,613 |

*** p<0.01. ** p<0.05. * p<0.1.

^1^The values for study year 2015-2016 are high in the logit regression because there were few zero observations.

**Table S4: Results of hurdle regressions on primary care consultations among males; adjusted for, age, education, marital status, geographical region, smoking status, and study period.**

| Variables | Logit  OR (SE) | GLM  IRR (SE) |
| --- | --- | --- |
| Underweight | 0.60 (0.17)* | 0.88 (0.24) |
| Overweight | 1.14 (0.06)*** | 1.12 (0.05)** |
| Category I obesity | 1.57 (0.14)*** | 1.52 (0.10)*** |
| Category II/III obesity | 2.22 (0.49)*** | 2.45 (0.24)*** |
| Age | 0.98 (0.01)*** | 0.99 (0.19) |
| Age^2 | 1.00 (0.00)*** | 1.01 (0.01) |
| Study period 2005-2006 | 0.74 (0.04)*** | 1.00 (0.00) |
| Study period 2008-2009 | 1.47 (0.09)*** | 1.06 (0.07) |
| Study period 2012-2013 | 1.59 (0.15)*** | 1.02 (0.06) |
| Study period 2015-2016^1^ | 82.63 (24.19)*** | 0.94 (0.08) |
| Higher education. long | 0.67 (0.06)*** | 1.01 (0.06) |
| Higher education. short | 0.83 (0.07)** | 0.62 (0.05)*** |
| Upper secondary education | 0.96 (0.06) | 0.68 (0.04)*** |
| Divorced /separated | 0.97 (0.09) | 0.84 (0.04)*** |
| Unmarried | 0.82 (0.05)*** | 1.30 (0.10)*** |
| Widow/widower | 0.84 (0.15) | 1.14 (0.06)** |
| Mid-North Norway | 0.90 (0.05)* | 1.11 (0.12) |
| Southern Norway | 0.93 (0.06) | 0.90 (0.05)** |
| Western Norway | 0.91 (0.06) | 0.96 (0.06) |
| Smoking daily | 1.09 (0.06) | 0.91 (0.05)* |
| Smoking occasionally | 1.06 (0.08) | 1.24 (0.07)*** |
| Constant | 3.08 (0.63)*** | 0.98 (0.07) |
| Number of observations | 13,155 | 10,294 |

*** p<0.01. ** p<0.05. * p<0.1.

^1^The values for study period 2015-2016 are high in the logit regression because there were few zero observations.

**Table S5: Results of hurdle regressions on primary care consultations among females; adjusted for, age, education, marital status, geographical region, smoking status, and study period.**

| Variables | Logit  OR (SE) | GLM  IRR (SE) |
| --- | --- | --- |
| Underweight | 1.02 (0.15) | 1.19 (0.14) |
| Overweight | 1.39 (0.09)*** | 1.20 (0.04)*** |
| Category I obesity | 1.81 (0.22)*** | 1.35 (0.06)*** |
| Category II/III obesity | 2.74 (0.73)*** | 2.03 (0.23)*** |
| Age | 1.00 (0.01) | 1.00 (0.00) |
| Age^2 | 1.00 (0.00) | 1.00 (0.00) |
| Study period 2005-2006 | 0.83 (0.05)*** | 1.09 (0.05)* |
| Study period 2008-2009 | 2.05 (0.15)*** | 1.05 (0.04) |
| Study period 2012-2013 | 1.70 (0.19)*** | 0.98 (0.07) |
| Study period 2015-2016^1^ | 117.67 (44.83)*** | 1.07 (0.04) |
| Higher education. long | 0.86 (0.11) | 0.76 (0.06)*** |
| Higher education. short | 1.02 (0.09) | 0.77 (0.03)*** |
| Upper secondary education | 1.02 (0.07) | 0.83 (0.03)*** |
| Divorced /separated | 1.20 (0.11)* | 1.32 (0.07)*** |
| Unmarried | 1.17 (0.09) | 1.09 (0.05)** |
| Widow/widower | 1.40 (0.18)*** | 1.11 (0.06)* |
| Mid-North Norway | 0.98 (0.07) | 0.87 (0.03)*** |
| Southern Norway | 0.84 (0.06)** | 1.01 (0.04) |
| Western Norway | 0.90 (0.06) | 0.99 (0.04) |
| Smoking daily | 1.24 (0.08)*** | 1.26 (0.05)*** |
| Smoking occasionally | 1.09 (0.09) | 0.96 (0.05) |
| Constant | 2.20 (0.53)*** | 3.24 (0.43)*** |
| Number of observations | 13,264 | 11,319 |

*** p<0.01. ** p<0.05. * p<0.1.

^1^The values for study period 2015-2016 are high in the logit regression because there were few zero observations.

**Table S6: Results of hurdle regressions on secondary care consultations; adjusted for gender, age, education, marital status, geographical region, smoking status, and study period.**

| Variables | Logit  OR (SE) | GLM  IRR (SE) |
| --- | --- | --- |
| Underweight | 1.06 (0.14) | 1.98 (0.59)** |
| Overweight | 1.12 (0.04)*** | 1.11 (0.09) |
| Category I obesity | 1.38 (0.08)*** | 1.05 (0.11) |
| Category II/III obesity | 1.40 (0.17)*** | 1.50 (0.20)*** |
| Female | 1.62 (0.06)*** | 0.82 (0.06)** |
| Age | 0.99 (0.01)** | 0.98 (0.01) |
| Age^2 | 1.00 (0.00)*** | 1.00 (0.00) |
| Study period 2012 | 1.19 (0.07)*** | 1.31 (0.17)** |
| Study period 2015 | 1.10 (0.04)** | 1.72 (0.13)*** |
| Higher education. long | 1.09 (0.08) | 0.78 (0.11)* |
| Higher education. short | 1.14 (0.06)** | 0.75 (0.08)*** |
| Upper secondary education | 1.08 (0.05) | 0.85 (0.08) |
| Divorced /separated | 1.19 (0.08)*** | 1.35 (0.16)** |
| Unmarried | 0.92 (0.04)* | 1.04 (0.10) |
| Widow/widower | 0.77 (0.07)*** | 1.08 (0.21) |
| Mid-North Norway | 0.95 (0.04) | 0.94 (0.08) |
| Southern Norway | 0.93 (0.05) | 1.01 (0.10) |
| Western Norway | 0.94 (0.05) | 0.96 (0.11) |
| Smoking daily | 1.06 (0.05) | 1.10 (0.11) |
| Smoking occasionally | 1.08 (0.07) | 1.25 (0.19) |
| Constant | 0.49 (0.08)*** | 0.00 (0.00)*** |
| Number of observations | 14,261 | 6,376 |

*** p<0.01. ** p<0.05. * p<0.1.

**Table S7: Results of hurdle regressions on secondary care consultations among males; adjusted for age, education, marital status, geographical region, smoking status, and study period.**

| Variables | Logit  OR (SE) | GLM  IRR (SE) |
| --- | --- | --- |
| Underweight | 1.32 (0.41) | 0.56 (0.25) |
| Overweight | 1.12 (0.06)** | 1.04 (0.11) |
| Category I obesity | 1.41 (0.12)*** | 1.05 (0.17) |
| Category II/III obesity | 1.87 (0.31)*** | 1.51 (0.33)* |
| Female | 0.98 (0.01)** | 1.00 (0.02) |
| Age | 1.00 (0.00)*** | 1.00 (0.00) |
| Age^2 | 1.22 (0.10)** | 1.35 (0.25) |
| Study period 2012-2013 | 1.13 (0.06)** | 1.69 (0.17)*** |
| Study period 2015-2016 | 0.98 (0.10) | 0.78 (0.16) |
| Higher education. long | 1.02 (0.08) | 0.72 (0.13)* |
| Higher education. short | 1.03 (0.07) | 0.87 (0.11) |
| Upper secondary education | 1.21 (0.12)* | 1.31 (0.22) |
| Divorced /separated | 1.00 (0.07) | 1.24 (0.18) |
| Unmarried | 0.87 (0.13) | 1.78 (0.68) |
| Widow/widower | 0.94 (0.06) | 0.83 (0.10) |
| Mid-North Norway | 0.97 (0.07) | 1.01 (0.15) |
| Southern Norway | 0.99 (0.07) | 0.75 (0.10)** |
| Western Norway | 1.05 (0.07) | 1.24 (0.19) |
| Smoking daily | 1.09 (0.10) | 1.29 (0.30) |
| Smoking occasionally | 0.45 (0.10)*** | 0.00 (0.00)*** |
| Constant | 1.32 (0.41) | 0.56 (0.25) |
| Number of observations | 7,094 | 2,780 |

*** p<0.01. ** p<0.05. * p<0.1.

**Table S8: Results of hurdle regressions on secondary care consultations among females; adjusted for age, education, marital status, geographical region, smoking status, and study period.**

| Variables | Logit  OR (SE) | GLM  IRR (SE) |
| --- | --- | --- |
| Underweight | 1.02 (0.15) | 2.44 (0.79)*** |
| Overweight | 1.16 (0.06)*** | 1.14 (0.11) |
| Category I obesity | 1.36 (0.12)*** | 1.00 (0.13) |
| Category II/III obesity | 1.11 (0.18) | 1.48 (0.24)** |
| Age | 0.99 (0.01) | 0.97 (0.02)* |
| Age^2 | 1.00 (0.00)* | 1.00 (0.00) |
| Study period 2012-2013 | 1.16 (0.10)* | 1.27 (0.19) |
| Study period 2015-2016 | 1.06 (0.06) | 1.69 (0.16)*** |
| Higher education. long | 1.17 (0.12) | 0.69 (0.12)** |
| Higher education. short | 1.20 (0.08)*** | 0.72 (0.09)** |
| Upper secondary education | 1.12 (0.07)* | 0.78 (0.10)* |
| Divorced /separated | 1.19 (0.10)** | 1.38 (0.22)** |
| Unmarried | 0.85 (0.06)** | 0.97 (0.12) |
| Widow/widower | 0.85 (0.09) | 0.80 (0.15) |
| Mid-North Norway | 0.96 (0.06) | 1.01 (0.12) |
| Southern Norway | 0.90 (0.07) | 0.95 (0.12) |
| Western Norway | 0.90 (0.06) | 1.11 (0.16) |
| Smoking daily | 1.06 (0.07) | 0.98 (0.12) |
| Smoking occasionally | 1.09 (0.09) | 1.19 (0.20) |
| Constant | 0.81 (0.17) | 0.00 (0.00)*** |
| Number of observations | 7,167 | 3,596 |

*** p<0.01. ** p<0.05. * p<0.1.
